# Multisystem Phenotypic Spectrum in Pediatric Heterotaxy Syndrome: A Case Series

**DOI:** 10.7759/cureus.91434

**Published:** 2025-09-01

**Authors:** Trisha G Mukherjee, Kyle E Thurmann, Randy R Richardson

**Affiliations:** 1 School of Medicine, Creighton University School of Medicine, Phoenix, USA; 2 Department of Radiology, Creighton University School of Medicine, Phoenix, USA

**Keywords:** atrial isomerism, congenital heart defects, heterotaxy syndrome, multisystem anomalies, pulmonary agenesis

## Abstract

Heterotaxy syndrome is a congenital condition characterized by abnormal left-right axis patterning of thoracoabdominal organs, frequently accompanied by complex congenital heart disease and extracardiac anomalies. The condition demonstrates considerable phenotypic variability, posing diagnostic and management challenges. We report three pediatric cases of heterotaxy syndrome classified by atrial isomerism: two with left atrial isomerism (LAI) and one with right atrial isomerism (RAI). The first case involved a 22-month-old male with LAI and polysplenia who presented with a transitional atrioventricular septal defect (AVSD), total anomalous pulmonary venous connection (TAPVC), Mobitz type II heart block, and a persistent gastrocutaneous fistula. He underwent staged surgical repair including TAPVC and AVSD correction, pulmonary valve commissurotomy, and pacemaker placement, and remained clinically stable. The second case involved a female neonate with RAI and asplenia, who presented at three weeks of age with heart failure and failure to thrive. She had complex cyanotic heart disease including double outlet right ventricle, unbalanced AVSD, TAPVC, and supraventricular tachycardia, along with extracardiac anomalies such as intestinal malrotation, gastrostomy tube dependence, and a lipomyelomeningocele. She was stabilized on medical therapy (furosemide, amlodipine, propranolol, and amoxicillin prophylaxis) and discharged home, later requiring readmission at seven months for parainfluenza infection but recovering well. At the latest follow-up, she remained clinically stable on medical management with plans for future biventricular repair. The third case involved a neonate with LAI and polysplenia who presented at birth with profound respiratory distress due to complete agenesis of the left lung, including absence of the left bronchus, pulmonary artery, and pulmonary veins, along with right pulmonary hypoplasia. Additional anomalies included a bifid right thumb. He received supportive care and was discharged on home oxygen. Genetic testing was non-diagnostic in all three patients despite extensive phenotypic abnormalities. Although the cases share hallmark features such as AVSD and abnormal pulmonary venous return, each demonstrates a distinct anatomic and clinical profile. This case series underscores the heterogeneity of heterotaxy syndrome and highlights the importance of comprehensive anatomic evaluation and multidisciplinary management.

## Introduction

Heterotaxy syndrome is a rare congenital disorder, occurring in approximately one per 10,000 live births, with associated congenital heart defects contributing significantly to neonatal and infant morbidity and mortality [1,2]. It is characterized by abnormal left-right axis patterning of thoracoabdominal organs, resulting from disruptions in embryonic development around the fifth week of gestation [1]. These disruptions stem from early errors in left-right patterning that are regulated by coordinated ciliary motion and downstream molecular signaling pathways [3-5]. Malfunctions in these processes can lead to both cardiac and extracardiac anomalies affecting multiple organ systems [5,6]. Although genes such as *ZIC3* and *NODAL* have been implicated, most cases lack a definitive molecular diagnosis, underscoring the ongoing need for phenotype-driven clinical assessment and research [3-5].

Clinically, heterotaxy syndrome is often classified based on spleen morphology or atrial appendage anatomy [2]. However, recent studies have highlighted significant anatomic variability and questioned the reliability of these traditional classification systems [1-3]. For the purpose of this case series, we adopt a classification based primarily on atrial isomerism, as defined by the morphology and symmetry of the atrial appendages [1,6]. In this system, patients are categorized as having right atrial isomerism (RAI) syndrome, typically associated with asplenia and more severe cardiac malformations; or left atrial isomerism (LAI) syndrome, more commonly linked to polysplenia and a broader range of extracardiac anomalies [2,7]. Although this classification has limitations, it provides a practical approach to discussing the phenotypic variability observed in heterotaxy syndrome.

This case series presents three pediatric cases of heterotaxy syndrome, each demonstrating unique combinations of cardiac and extracardiac anomalies. By comparing clinical presentations across isomerism subtypes, this series highlights the heterogeneity of the condition and underscores the importance of tailored, anatomy-based approaches to diagnosis and management.

## Case presentation

The following case series illustrates the clinical diversity of heterotaxy syndrome through three pediatric patients, including two with LAI and one with RAI. Despite shared features such as atrioventricular septal defects (AVSDs) and abnormal pulmonary venous connections, each case is distinguished by unique anatomic findings, extracardiac manifestations, and management strategies. A comparative overview of clinical features is provided in Table 1, followed by detailed individual case descriptions.

**Table 1:** Comparative summary of clinical, anatomic, and management features in three pediatric patients with heterotaxy syndrome This table compares three pediatric patients with heterotaxy syndrome, highlighting differences in age, subtype classification, cardiac and extracardiac anomalies, interventions, and clinical outcomes. Laterality is reported based on atrial isomerism and associated splenic morphology. Each row outlines key phenotypic features used in diagnosis and management. AC/VC: assist control/volume control ventilation; ASD: atrial septal defect; AV: atrioventricular; AVSD: atrioventricular septal defect; DORV: double outlet right ventricle; G-tube: gastrostomy tube; HFOV: high-frequency oscillatory ventilation; LAI: left atrial isomerism; LSVC: left superior vena cava; LV: left ventricle; MR: mitral regurgitation; NIPPV: noninvasive positive pressure ventilation; PDA: patent ductus arteriosus; PR: pulmonary regurgitation; PS: pulmonary stenosis; RAI: right atrial isomerism; RSVC: right superior vena cava; RV: right ventricle; SVT: supraventricular tachycardia; TAPVC: total anomalous pulmonary venous connection; TR: tricuspid regurgitation; VSD: ventricular septal defect.

Patient characteristics	Case 1	Case 2	Case 3
Sex/Age at Presentation	Male, 22 months	Female, neonate (at birth)	Male, neonate (at birth)
Heterotaxy Subtype	LAI (polysplenia)	RAI (asplenia)	LAI (polysplenia)
Laterality/Situs	Situs ambiguous (presumed), midline liver, bilateral left-sided tracheobronchial branching	Situs ambiguous, levocardia, midline liver, right-sided stomach, bilateral right-sided tracheobronchial branching	Situs ambiguous, midline liver, left-sided gallbladder
Cardiac Anomalies	Traditional AVSD (large primum ASD, small VSD, mitral valve cleft); TAPVC; severe valvular PS; persistent LSVC; unroofed coronary sinus; Mobitz type II second-degree AV block	DORV with D-malposed great arteries; complete unbalanced AVSD (RV dominance); large inlet-to-outlet VSD; TAPVC to RSVC; persistent LSVC; mildly hypoplastic LV; severe PS; mild common AV valve insufficiency; SVT	Large PDA; small ASD; elevated RV pressures; hypoplastic right pulmonary artery and lung; absent left pulmonary artery and lung
Extracardiac Anomalies	Persistent gastrocutaneous fistula	Intestinal malrotation; gastrostomy tube dependence; lipomyelomeningocele	Complete agenesis of left lung, bronchus, and pulmonary artery; bifid right thumb; mild left renal pelvic fullness
Interventions	TAPVC repair; AVSD repair with autologous pericardial patch; mitral cleft closure; pulmonary valve commissurotomy; LSVC ligation; dual-chamber epicardial pacemaker	Medical management: furosemide, amlodipine, propranolol, amoxicillin prophylaxis; planned biventricular repair	Intubation at birth; chest tube placement for pneumothorax; mechanical ventilation (HFOV → AC/VC → NIPPV → nasal cannula); supportive management only, no surgical intervention to date
Clinical Status	Mild residual TR, MR, and PR; improved PS gradient; good biventricular function; not pacemaker dependent; sinus bradycardia; clinically stable and developmentally appropriate	Oxygenating well on room air; gaining weight with G-tube feeds; no recent hospitalizations; stable on medical regimen; undergoing surveillance for planned biventricular repair	Discharged on home nasal cannula with pulse oximetry; no pulmonary hypertension; stable clinical course at discharge; feeding plan and immunizations complete

Case 1

**Figure 1:**
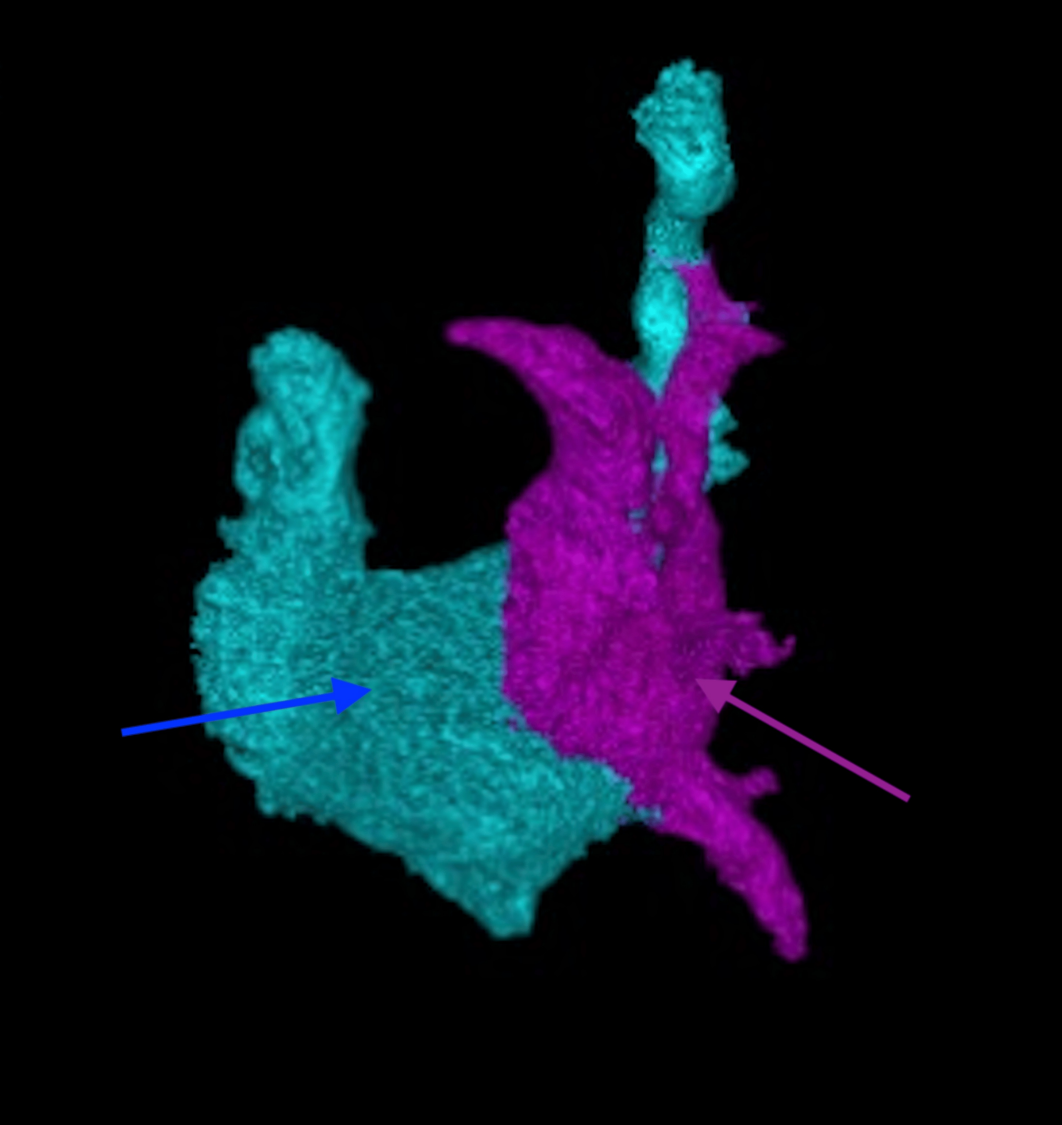
Three-dimensional computed tomography reconstruction demonstrating bilateral symmetric left-sided atrial appendages (frontal oblique view) The blue arrow identifies the left atrial appendage and the purple arrow highlights the right atrial appendage, which mirrors the left in shape and orientation.

**Figure 2:**
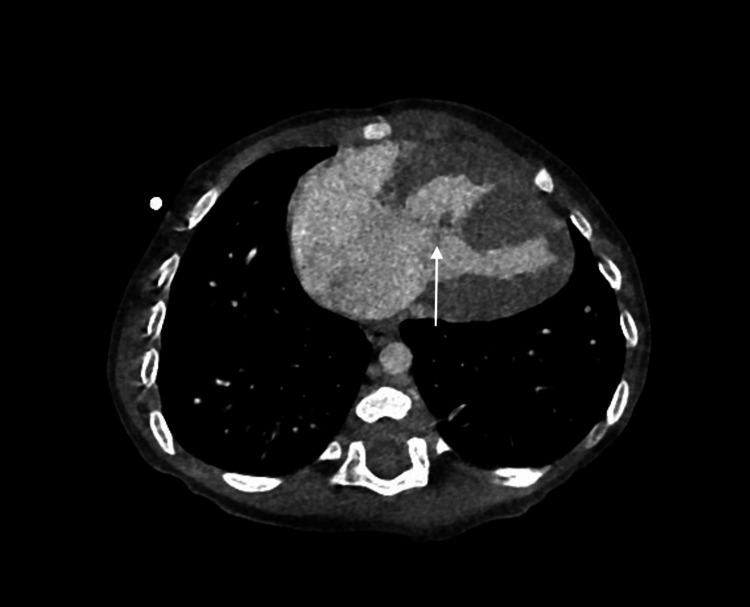
Axial contrast-enhanced computed tomography demonstrating a transitional atrioventricular septal defect The white arrow indicates absence of the atrioventricular septum, with a common atrioventricular junction.

**Figure 3:**
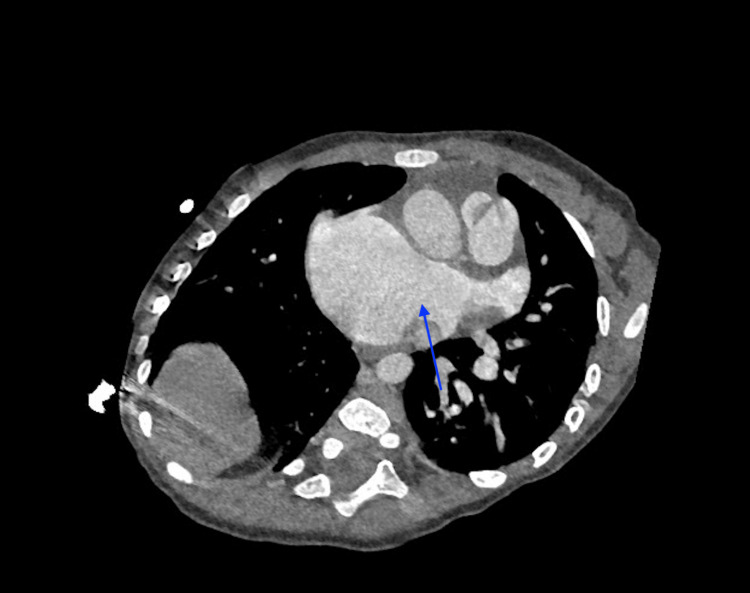
Axial contrast-enhanced computed tomography image demonstrating a large primum atrial septal defect (blue arrow)

**Figure 4:**
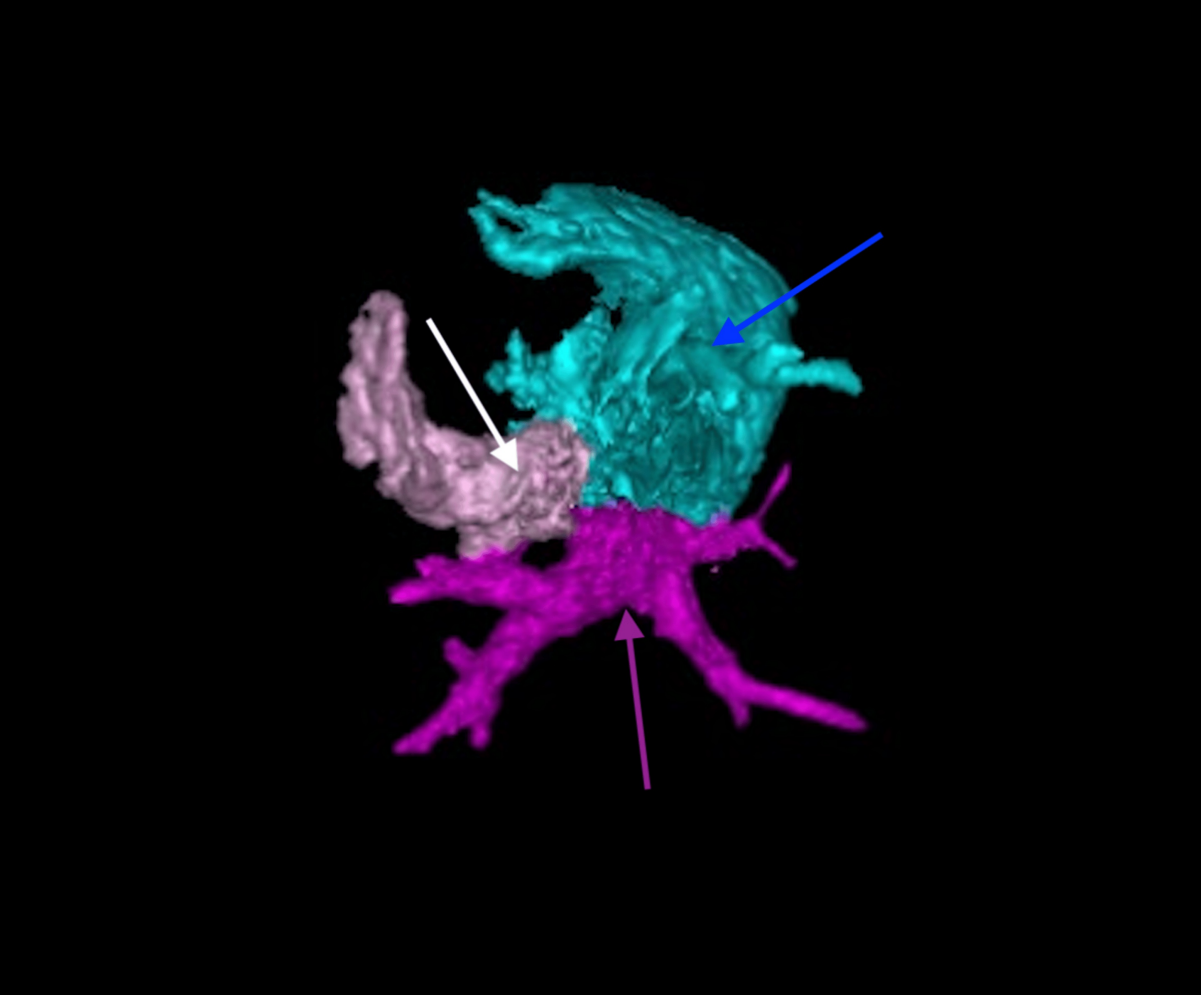
Three-dimensional computed tomography reconstruction demonstrating total anomalous pulmonary venous connection with common atrial drainage and an unroofed coronary sinus (superior view) The blue arrow indicates the anomalous pulmonary veins, the white arrow shows the common venous confluence and unroofed coronary sinus, and the purple arrow highlights the systemic venous drainage via the persistent left superior vena cava.

**Figure 5:**
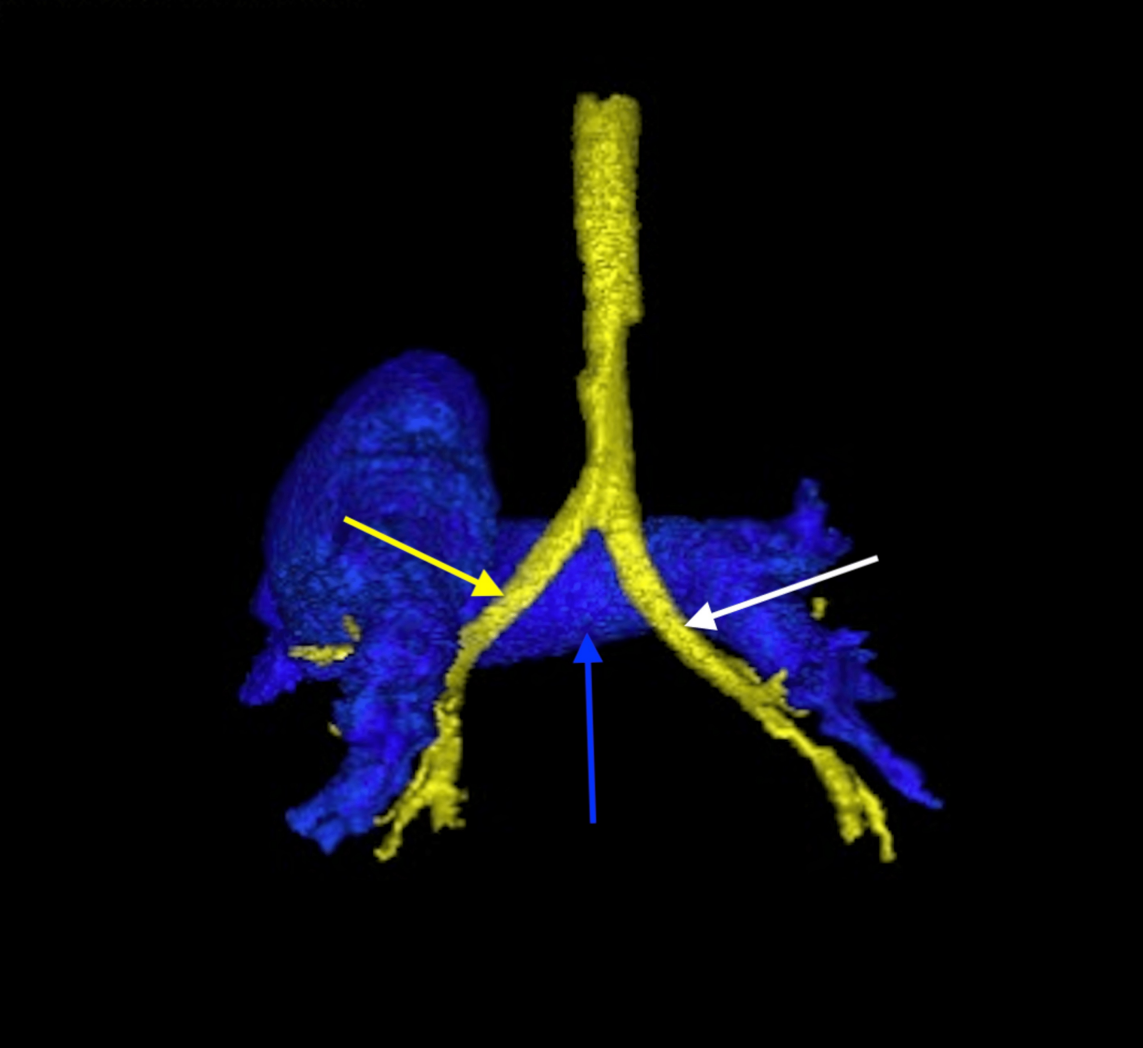
Three-dimensional computed tomography reconstruction demonstrating bilateral left-sided tracheobronchial branching (posterior view) The yellow arrow indicates the left main bronchus with normal left-sided branching morphology. The white arrow identifies the right main bronchus, which mirrors the left in its horizontal orientation, consistent with left-sided morphology. The blue arrow marks the main pulmonary artery, which lies anterior to the tracheal bifurcation.

A 22-month-old male with heterotaxy syndrome consistent with LAI presented with multiple congenital anomalies. These included bilateral symmetric left-sided atrial appendages (Figure 1), transitional AVSD (Figure 2) with a large primum atrial septal defect (ASD; Figure 3), small ventricular septal defect (VSD), mitral valve cleft, total anomalous pulmonary venous connection (TAPVC; Figure 4) with common atrial drainage, severe valvular pulmonary stenosis (PS), persistent left superior vena cava (LSVC; Figure 4), unroofed coronary sinus (Figure 4), bilateral left-sided tracheobronchial branching (Figure 5), Mobitz type II second-degree atrioventricular (AV) block, and polysplenia. He underwent intracardiac TAPVC repair, AVSD repair with autologous pericardial patch and mitral cleft closure, pulmonary valve commissurotomy, LSVC ligation, and placement of a dual-chamber epicardial pacemaker. Postoperative recovery was notable for a right-sided pleural effusion requiring chest tube placement, which resolved by postoperative day five. Follow-up echocardiograms demonstrated good biventricular function, no residual VSD, mild residual valvular regurgitation, and improvement in the PS gradient (from 45 mmHg preoperatively to 13 mmHg at latest follow-up). He was not pacemaker-dependent and maintained sinus bradycardia. A persistent gastrocutaneous fistula from a prior gastrostomy tube remained; however, surgical repair was deferred due to its proximity to the pacemaker. At over one year postoperatively, he remains clinically stable, well perfused, and developmentally appropriate, without cyanosis or exertional symptoms.

Case 2

**Figure 6:**
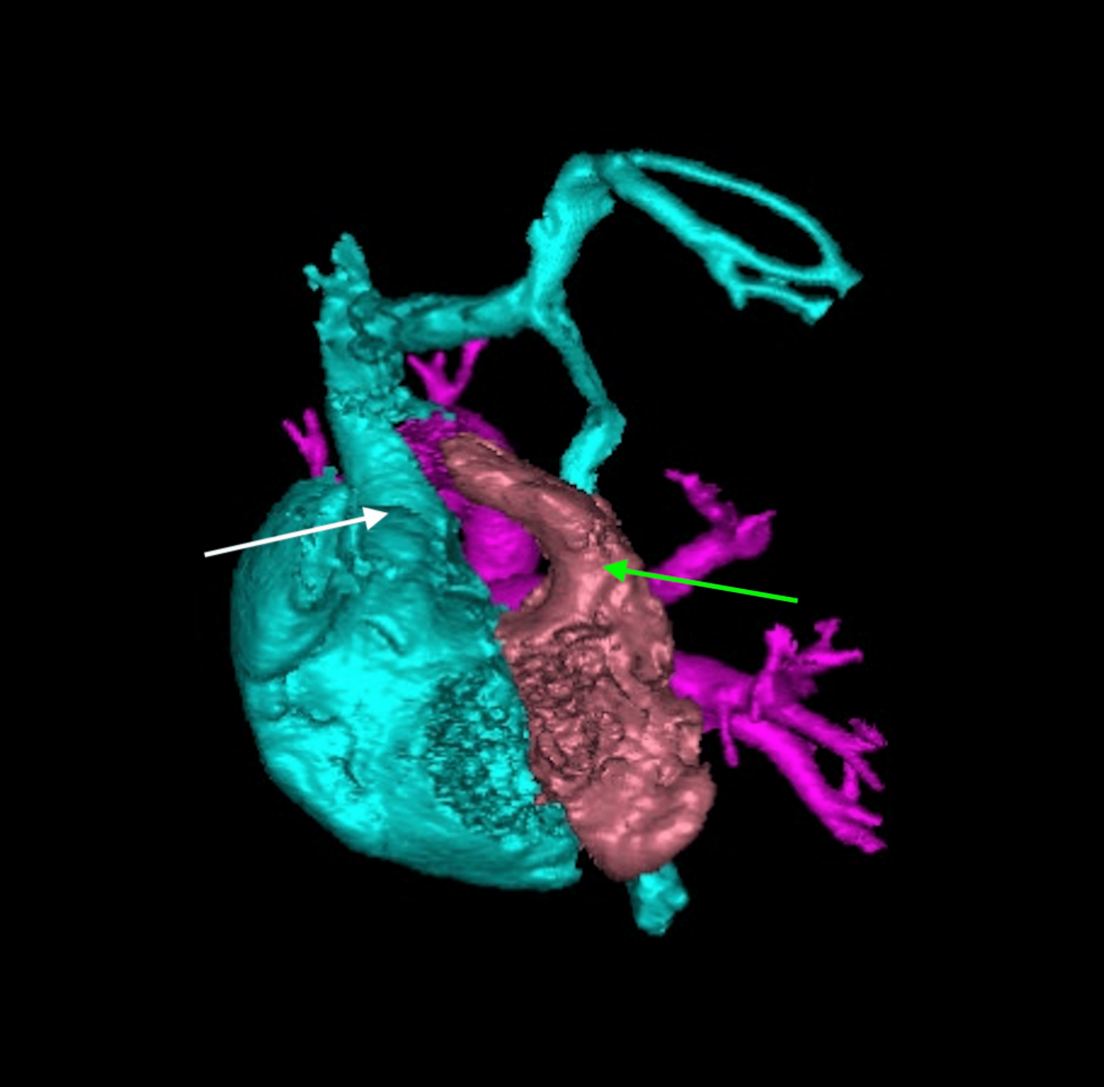
Three-dimensional computed tomography reconstruction demonstrating symmetric right atrial appendages (frontal oblique view) The white arrow indicates the right atrial appendage, and the green arrow identifies the left atrial appendage, which mirrors the right in its broad, triangular configuration.

**Figure 7:**
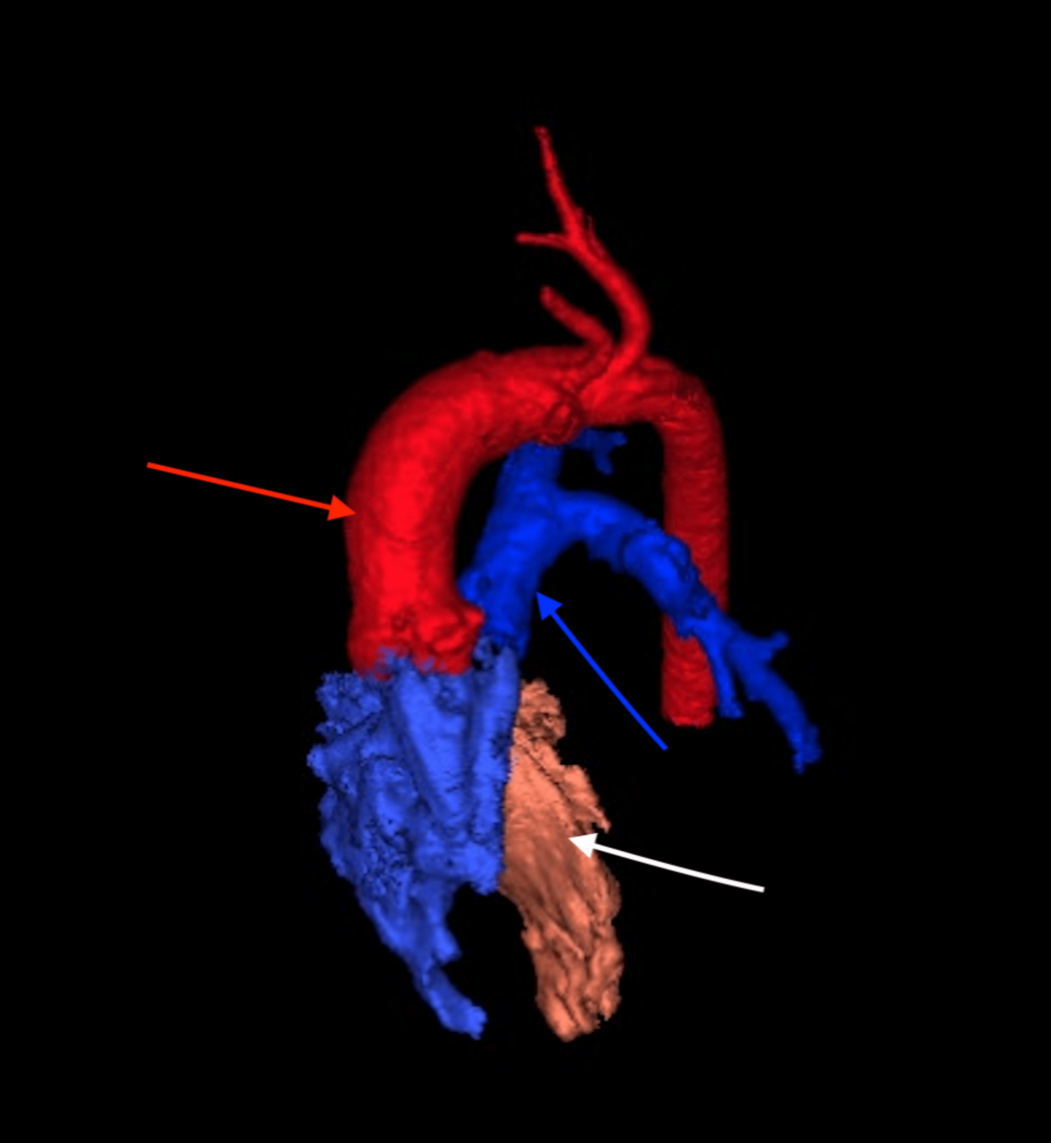
Three-dimensional computed tomography reconstruction demonstrating double outlet right ventricle with D-malposed great arteries (anterior superior view) The red arrow indicates the aorta, which arises anteriorly and rightward from the right ventricle. The blue arrow marks the malposed pulmonary artery, arising posteriorly and leftward. The white arrow identifies the right ventricle, from which both great arteries originate.

**Figure 8:**
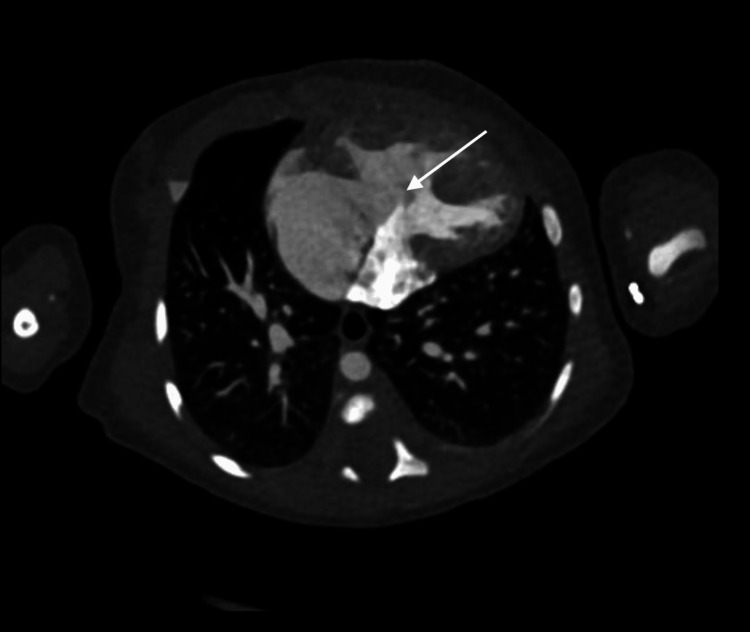
Axial contrast-enhanced computed tomography image demonstrating a complete, unbalanced atrioventricular septal defect with right ventricular dominance The white arrow highlights the common atrioventricular junction overriding a hypoplastic left ventricle, consistent with right ventricular dominance.

**Figure 9:**
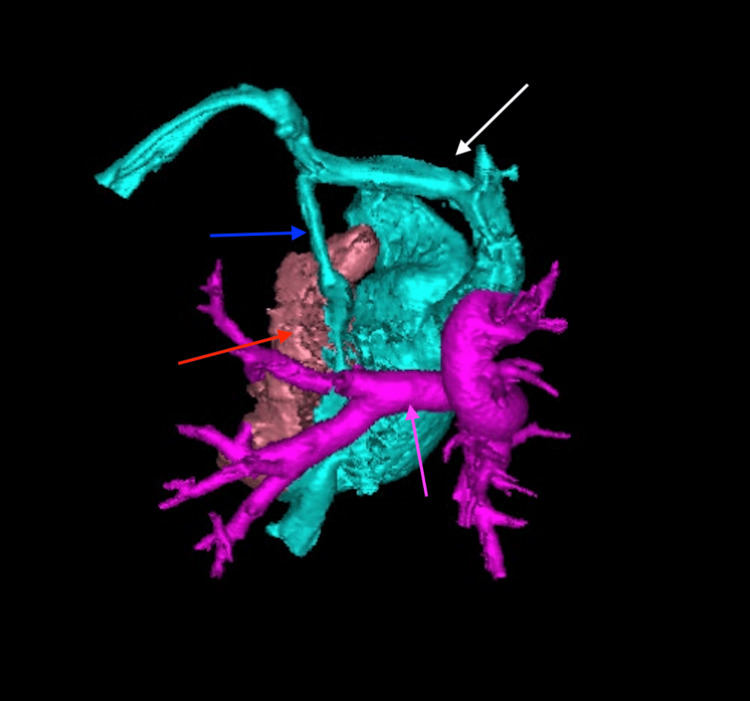
Three-dimensional computed tomography angiography reconstruction (posterior view) showing bilateral superior vena cavae: right superior vena cava (white arrow) and persistent left superior vena cava (blue arrow) The pulmonary venous confluence is indicated by the red arrow. The main pulmonary artery (pink arrow) arises from the right ventricle.

**Figure 10:**
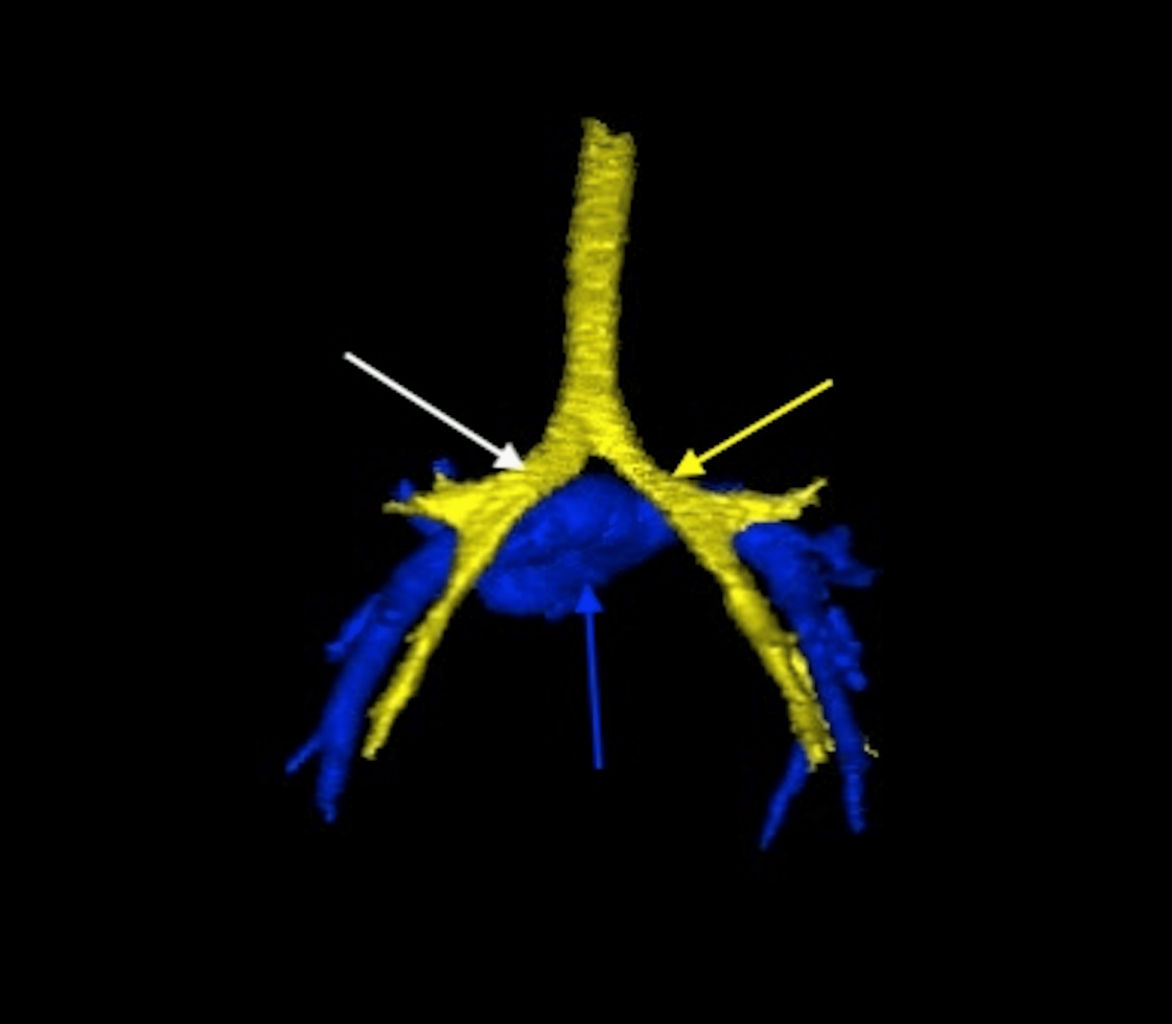
Three-dimensional computed tomography reconstruction demonstrating bilateral right-sided tracheobronchial branching (posterior view) The yellow arrow indicates the right main bronchus with normal right-sided branching morphology. The white arrow identifies the left main bronchus, which mimics the right in its short and vertical course, consistent with right-sided morphology. The blue arrow marks the main pulmonary artery, which lies anterior to the carina.

**Figure 11:**
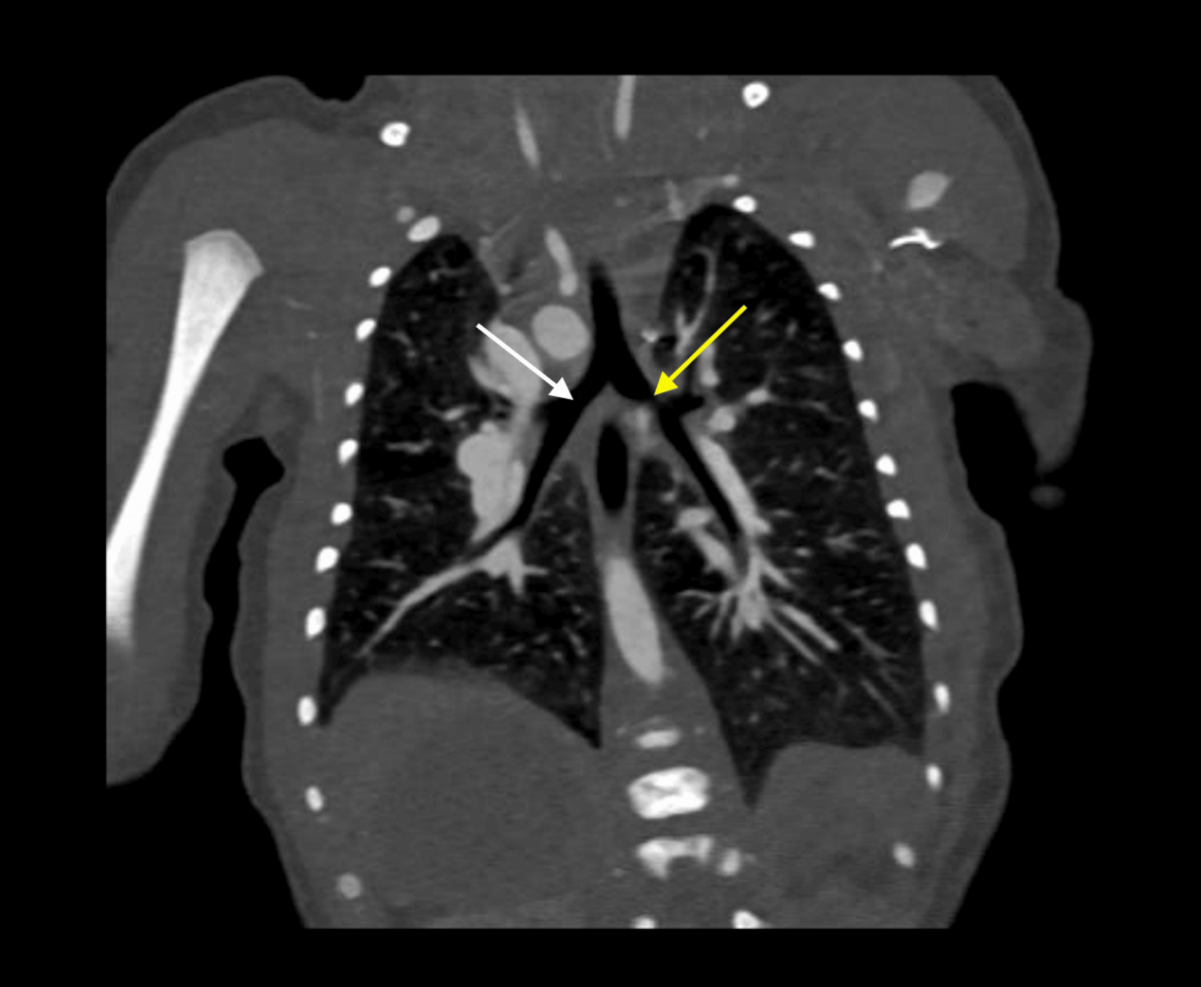
Coronal contrast-enhanced computed tomography image demonstrating bilateral right-sided tracheobronchial branching The yellow arrow identifies the right main bronchus with typical short and vertical right-sided morphology. The white arrow indicates the left main bronchus, which mirrors the right in its configuration, consistent with bilateral right-sided bronchial anatomy.

**Figure 12:**
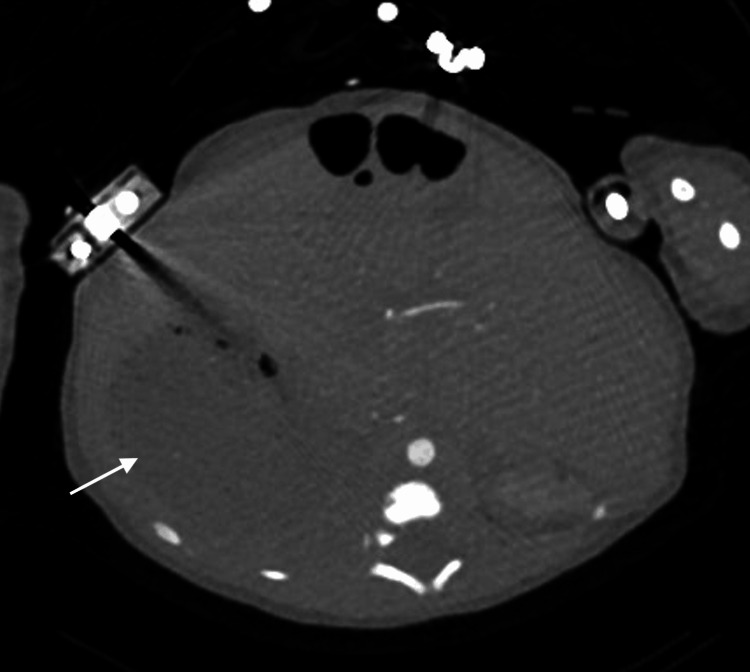
Axial contrast-enhanced computed tomography image demonstrating a right-sided stomach (white arrow)

A female neonate was born at 38 weeks' gestation via repeat cesarean section following a pregnancy complicated by prenatal diagnosis of heterotaxy and complex congenital heart disease. Her neonatal course was unremarkable, requiring no cardiac intervention, and she was discharged home on day nine of life. At three weeks of age, she was admitted to the pediatric care unit with symptoms of congestive heart failure and failure to thrive. Inpatient evaluation, including echocardiography and magnetic resonance imaging, identified multiple congenital anomalies, which were later confirmed and further characterized on computed tomography imaging. Cardiac anomalies included symmetric right atrial appendages (Figure 6), double outlet right ventricle (DORV) with D-malposed great arteries (Figure 7), complete unbalanced AVSD with right ventricular dominance (Figure 8), large inlet-to-outlet VSD, TAPVC to the right superior vena cava (Figure 9), persistent LSVC (Figure 9), mildly hypoplastic left ventricle, severe PS (peak gradient of 68 mmHg), and mild common AV valve insufficiency. Additional findings included situs ambiguous with levocardia, bilateral right-sided tracheobronchial branching (Figures 10, 11), asplenia, midline liver, right-sided stomach (Figure 12), congenital intestinal malrotation, gastrostomy tube dependence, lipomyelomeningocele, and supraventricular tachycardia. Once stabilized, she was discharged home on nasogastric/gastrostomy tube feeds and a regimen of furosemide, amlodipine, propranolol, and amoxicillin prophylaxis. At seven months of age, she was re-admitted with fever, vomiting, and feeding intolerance and tested positive for a parainfluenza virus infection. Due to her asplenia, she was treated empirically with intravenous (IV) antibiotics and was later discharged in stable condition. She continued to oxygenate well on room air with saturations greater than 85% and remained stable on her medical regimen. At the latest follow-up, she was tolerating her gastrostomy feeds, gaining weight appropriately, and remained under surveillance for planned biventricular repair at a pediatric surgical center.

Case 3

**Figure 13:**
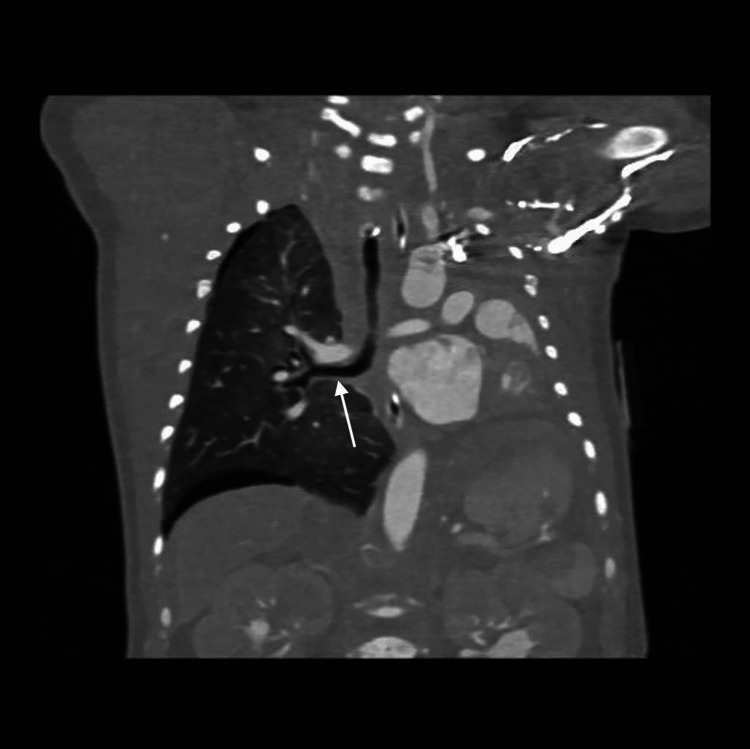
Coronal computed tomography image illustrating complete left lung agenesis with marked leftward mediastinal shift The left main bronchus is absent, and only the right main bronchus is visualized (white arrow), consistent with unilateral pulmonary agenesis.

**Figure 14:**
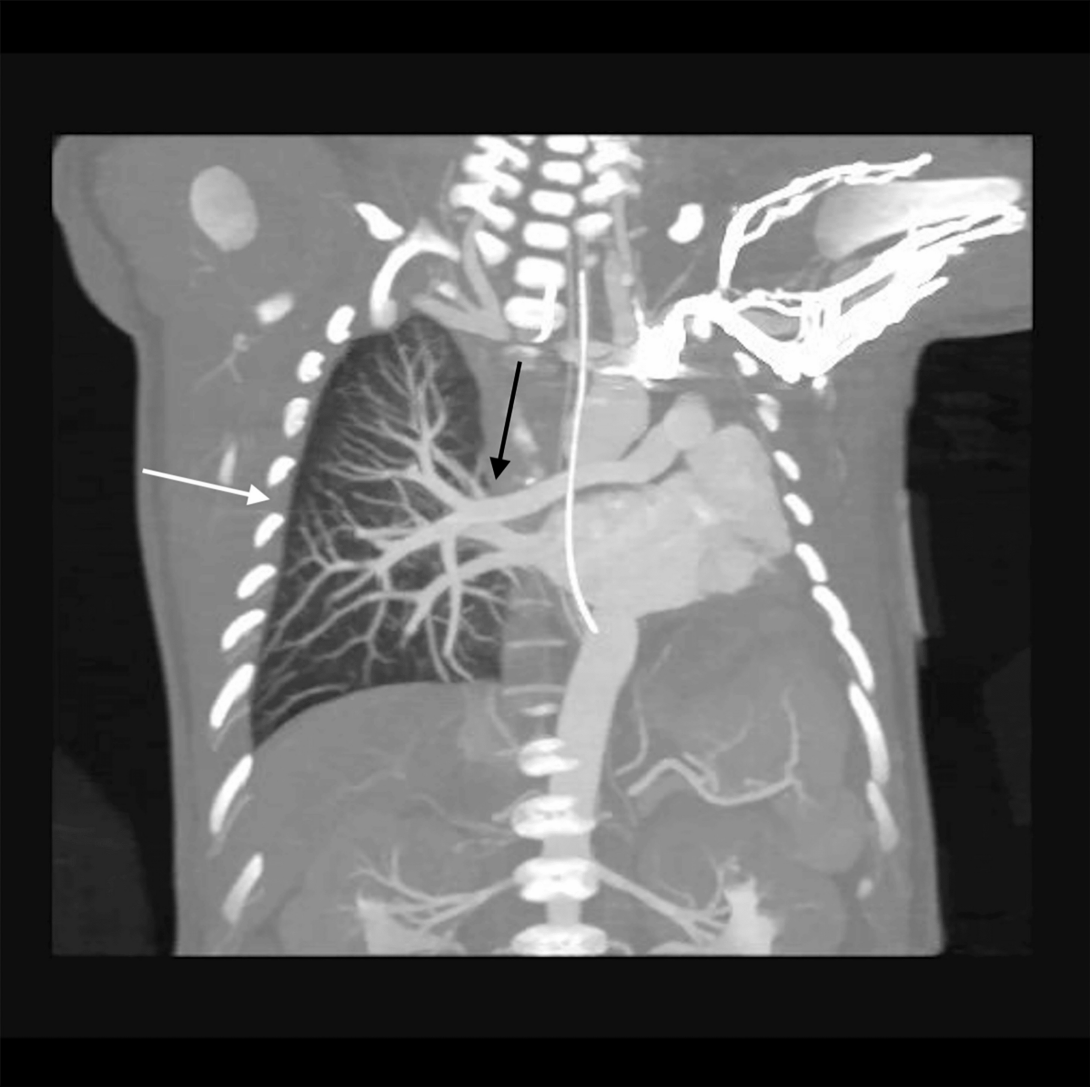
Coronal computed tomography angiography demonstrating complete left lung agenesis with absence of the left pulmonary artery and pulmonary veins The right lung (white arrow) is hyperexpanded and compensates by receiving the entire pulmonary arterial flow (black arrow).

**Figure 15:**
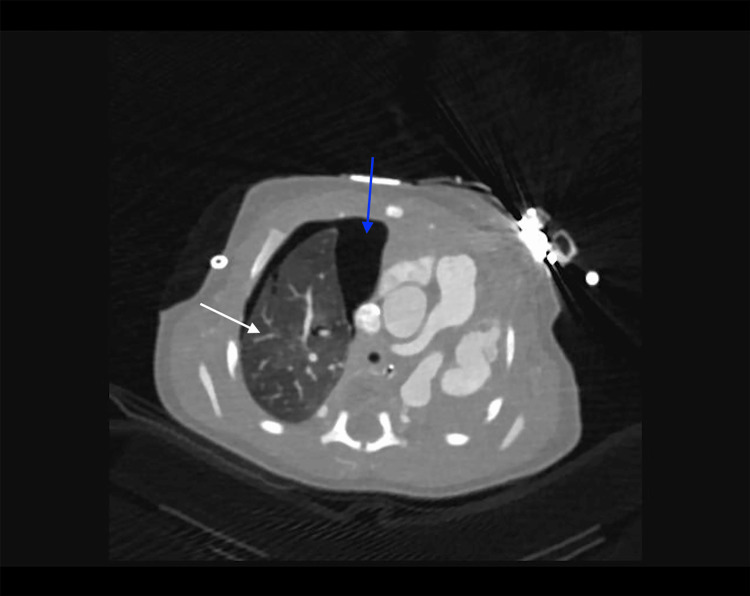
Axial computed tomography image showing complete agenesis of the left lung with associated mediastinal shift to the left The right lung (white arrow) is hyperexpanded and compensatory in appearance. A small right-sided pneumothorax is also present (blue arrow).

**Figure 16:**
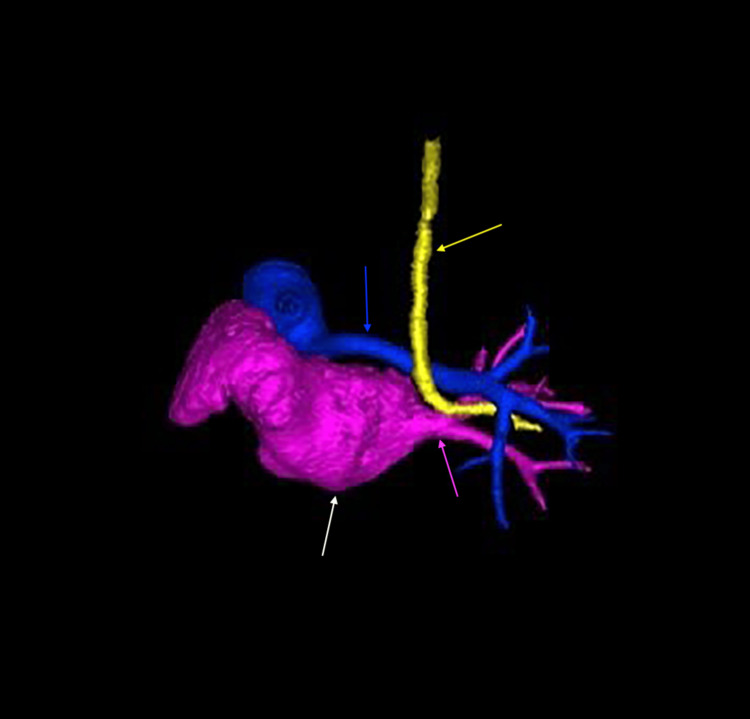
Three-dimensional computed tomography reconstruction demonstrating complete agenesis of the left lung with absence of the left pulmonary vasculature (posterior inferior view) Only the right pulmonary vessels are visualized, including the right pulmonary vein (pink arrow) and right pulmonary artery (blue arrow). The heart (white arrow) is positioned toward the left hemithorax, and the trachea is deviated (yellow arrow), reflecting a significant mediastinal shift.

**Figure 17:**
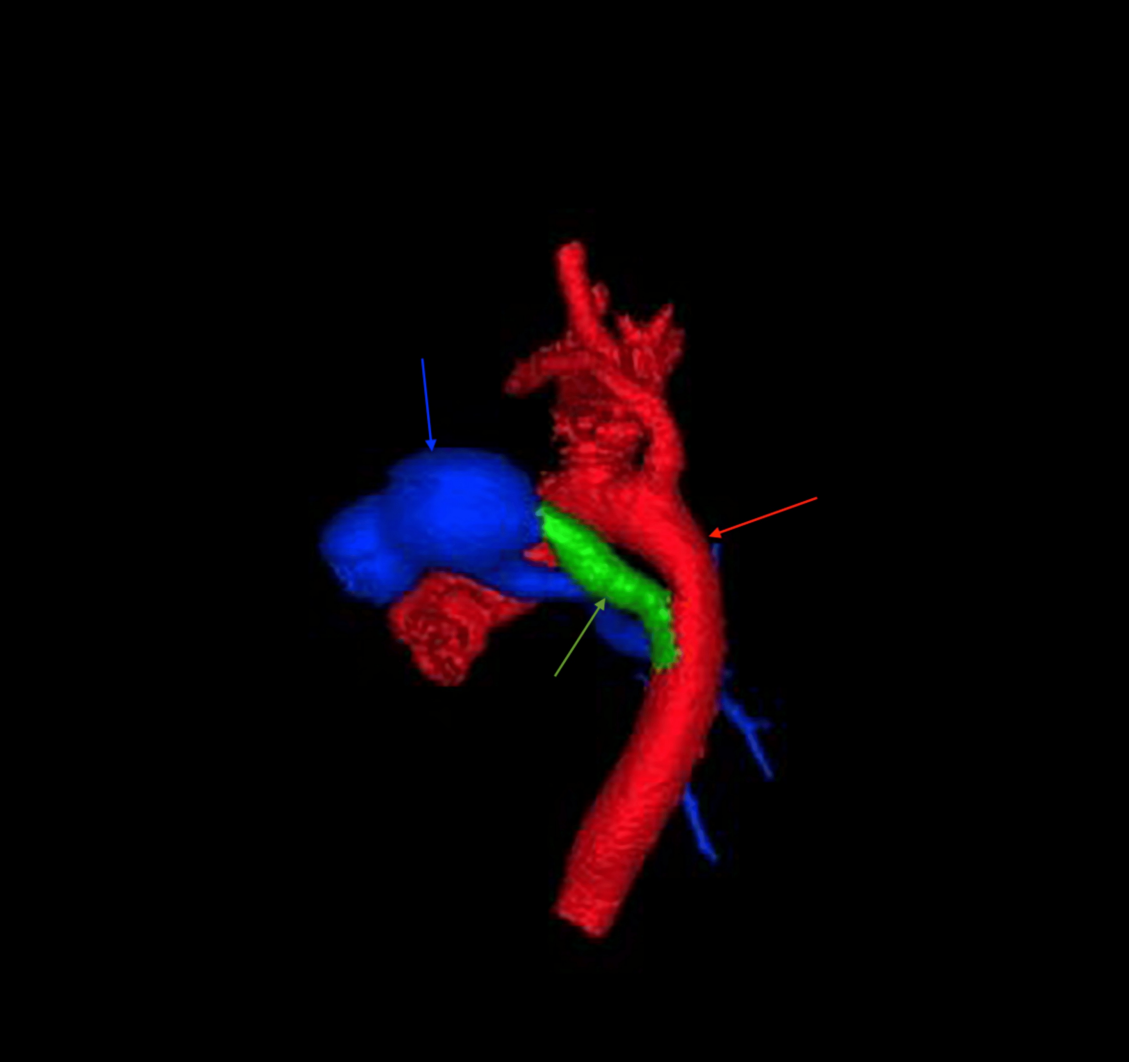
Three-dimensional computed tomography angiography reconstruction (left lateral view) demonstrating patent ductus arteriosus (green arrow) The patent ductus arteriosus connects the hypoplastic right pulmonary artery (blue arrow) to the descending thoracic aorta (red arrow).

**Figure 18:**
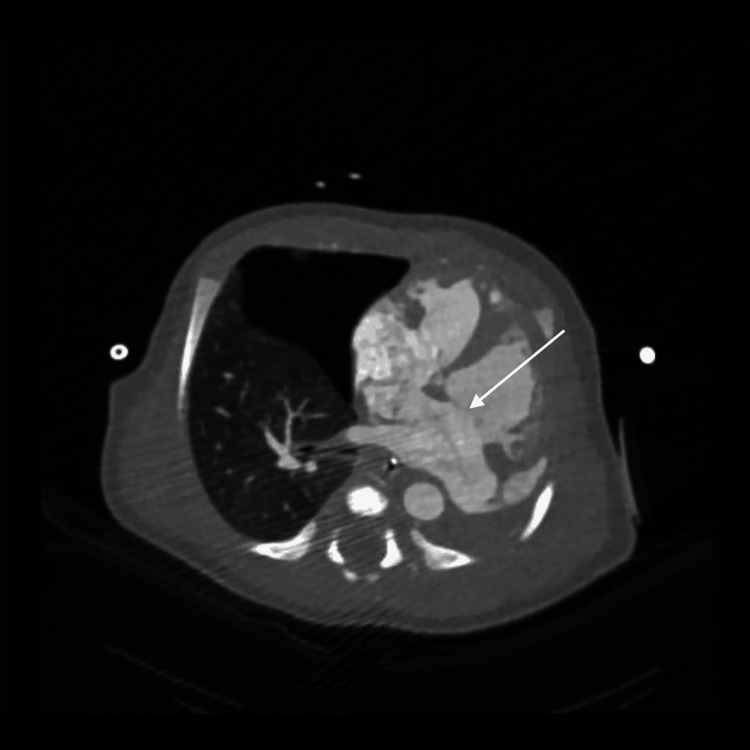
Axial computed tomography image revealing a communication across the interatrial septum consistent with an atrial septal defect (white arrow)

**Figure 19:**
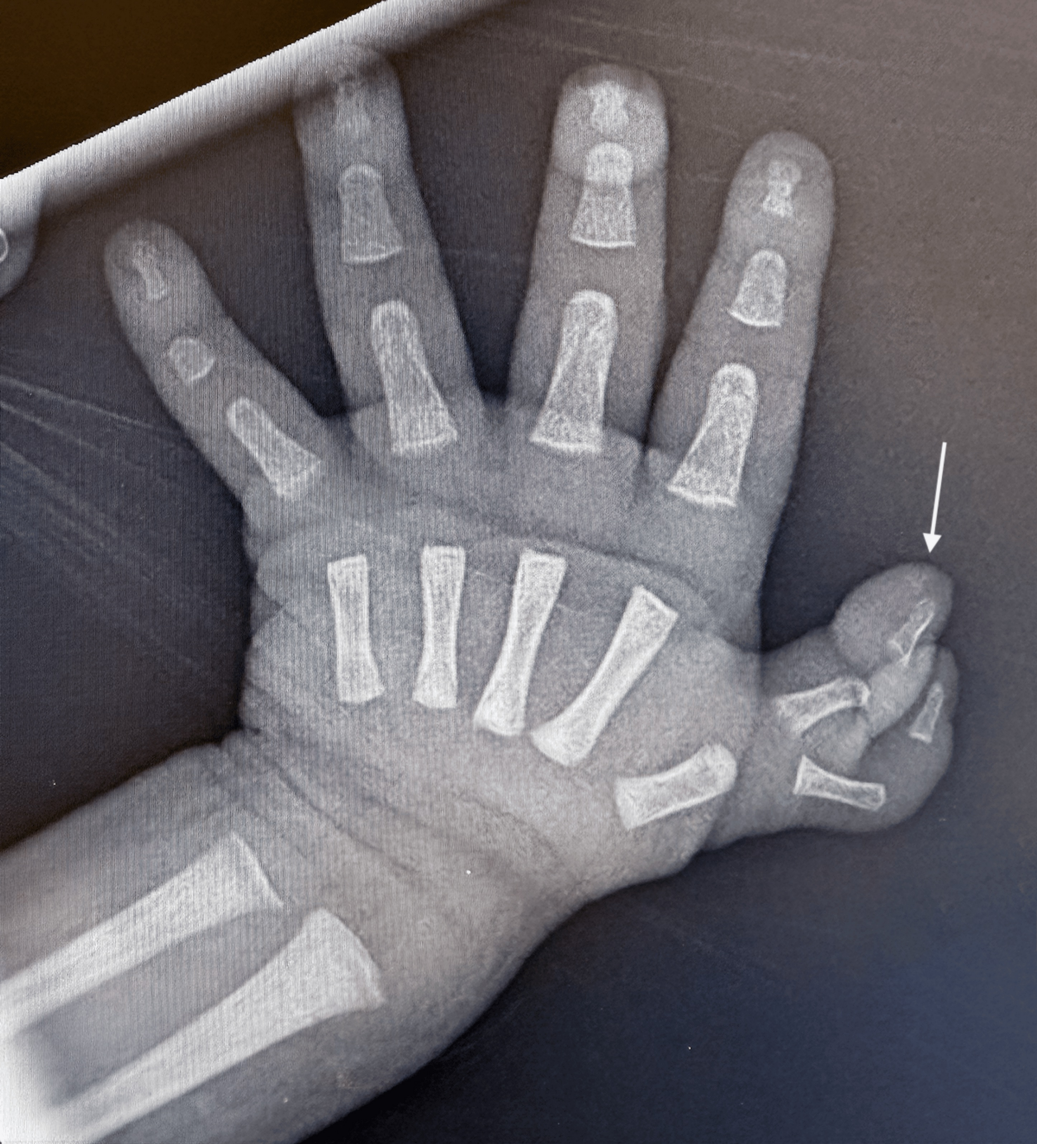
Anteroposterior radiograph of the right hand demonstrating a bifid thumb (white arrow)

A male neonate born at 39 weeks’ gestation with heterotaxy syndrome consistent with LAI presented at birth with severe respiratory distress, bradycardia, and cyanosis requiring intubation and mechanical ventilation. Initial chest imaging revealed complete opacification of the left hemithorax with mediastinal shift and an elevated gastric bubble. Further evaluation with echocardiography and chest computed tomography demonstrated complete agenesis of the left lung, left bronchus, left pulmonary artery, and left pulmonary veins (Figures 13-16). Additional findings included a large patent ductus arteriosus (Figure 17), a small ASD (Figure 18), and elevated right ventricular pressures. The right lung and right pulmonary artery were hypoplastic. Abdominal ultrasound revealed polysplenia, a midline liver, and a left-sided gallbladder. Other anomalies included mild left renal pelvic fullness and a bifid right thumb (Figure 19). Trio-based exome sequencing did not identify any pathogenic or likely pathogenic variants. The patient remained on ventilatory support throughout hospitalization and was discharged home on low-flow nasal cannula. Follow-up care was arranged with pulmonology, cardiology, genetics, and immunology.

## Discussion

Classification by laterality subtype

In this case series, two cases (cases 1 and 3) were classified as having LAI with polysplenia, and one case (case 2) had RAI with asplenia. These laterality subtypes, defined by both atrial morphology and splenic status, reflect well-established clinical patterns: RAI is typically associated with more severe cyanotic congenital heart disease, while LAI encompasses a broader range of structural and extracardiac anomalies [5,6,8,9].

Cardiac phenotypes

Despite heterogeneity in anatomy, all three cases shared hallmark cardiac features (Table 1). AVSDs were present in cases 1 and 2, consistent with the high prevalence of endocardial cushion defects in heterotaxy [10,11]. This reflects broader cohort data showing AVSDs in 59% of patients with LAI and 73% of those with RAI [10]. Case 3 had a small ASD without AV valve abnormalities. TAPVC, which is classically associated with RAI, was present in both cases 1 and 2 [5,9,12]. While case 2 had the expected RAI subtype, case 1’s TAPVC illustrates that this anomaly can also occur in LAI, though less commonly [9,12]. Its presence in this context suggests partial phenotypic overlap between LAI and RAI, particularly in systemic and pulmonary venous development [9,12]. In contrast, case 3 demonstrated a different mechanism of pulmonary venous abnormality: complete agenesis of the left pulmonary artery and pulmonary veins, representing pulmonary vascular agenesis, a rare developmental anomaly distinct from TAPVC [13].

Additional features followed known patterns by laterality subtype. Case 2 (RAI) had the most complex and cyanotic anatomy, including DORV, unbalanced AVSD, severe PS, supraventricular tachycardia (SVT), and a mildly hypoplastic left ventricle. Cases 1 and 3 (LAI) had a broader range of cardiac findings, including a Mobitz II second-degree AV block in case 1 and elevated right-sided pressures in case 3. Notably, the contrast between these two cases highlights the wide phenotypic spectrum seen in LAI, from complex AV canal and conduction anomalies to milder structural findings with abnormal pulmonary vasculature.

These findings reinforce the established association between RAI and severe conotruncal cardiac disease, while LAI more often presents with variable, and sometimes milder, structural anomalies [5,6,9].

Extracardiac findings

While cardiac anomalies are the most well-characterized feature of heterotaxy, extracardiac manifestations are also common and clinically significant [5,6,10]. In this series, each case exhibited distinct extracardiac findings affecting multiple organ systems.

Case 3 (LAI) had the most striking anomalies, including complete agenesis of the left lung, bronchus, and pulmonary artery, as well as hypoplasia of the right pulmonary artery and lung. Although rare, such severe pulmonary underdevelopment reflects the broader spectrum of laterality disturbances seen in heterotaxy and demonstrates the extent to which airway and vascular development can be affected [14]. Additional findings, such as a bifid right thumb and mild left renal pelvic fullness, suggest broader laterality disturbance beyond the cardiopulmonary system [9,15].

Case 2 (RAI) presented with a more classic extracardiac profile, including intestinal malrotation and gastrostomy tube dependence, both well-documented associations in RAI [6,12]. She also had a lipomyelomeningocele, a less common but documented co-occurrence in heterotaxy that points to possible shared pathways in neural and visceral patterning [16,17].

Case 1 (LAI) had the fewest extracardiac anomalies, with only a persistent gastrocutaneous fistula. His relatively mild extracardiac profile contrasts with the broader range of anomalies frequently described in LAI, highlighting the phenotypic variability within this isomerism subtype [2,6,7].

Together, these findings underscore the multisystem nature of heterotaxy syndrome, with involvement of the respiratory, gastrointestinal, genitourinary, and musculoskeletal systems [5,6,15].

Developmental considerations

The spectrum of anomalies in this cohort reflects disruptions in left-right axis formation during early embryogenesis [6]. Heterotaxy arises from dysregulation of molecular signaling pathways, including Sonic Hedgehog, Nodal, Lefty, and Pitx2, that are essential for establishing organ laterality and proper cardiac development [5,9,15]. Mutations in over 20 genes regulating these pathways have been linked to heterotaxy, with *ZIC3*, *NODAL*, and *CFC1* among the most commonly implicated [5]. This genetic heterogeneity contributes to the clinical variability seen across patients. Genetic testing was non-diagnostic in our series, highlighting current limitations in molecular diagnostics and reinforcing the continued importance of phenotype-driven evaluation, especially when anomalies span multiple organ systems [5,6,17].

Clinical course and management considerations

The management of heterotaxy syndrome requires a multidisciplinary approach, reflecting the broad anatomic and physiologic variability across patients [5,17]. In this series, clinical trajectories ranged from staged surgical repair to exclusively supportive care, underscoring the spectrum of complexity in both structural disease and medical decision-making.

Case 1 (LAI) underwent multiple surgical interventions, including TAPVC and AVSD repair, pulmonary valve commissurotomy, and pacemaker placement for Mobitz type II heart block. His relatively favorable postoperative course, with preserved biventricular function, mild residual valvular disease, and no pacemaker dependence, highlights how certain patients with LAI can achieve clinical stability following definitive intervention.

In contrast, case 2 (RAI) presented the most complex anatomy, including DORV, unbalanced AVSD, and severe PS. She was medically managed in infancy with diuretics and antihypertensives, with planned biventricular repair following medical optimization. Importantly, she was hospitalized with a parainfluenza infection and treated empirically with IV antibiotics, reflecting appropriate clinical concern for immune dysfunction associated with asplenia [12,17]. Although her infection was viral, her management illustrates the heightened vigilance required to mitigate risks of sepsis in this high-risk group [5,6,12,17].

Case 3 (LAI) presented at birth with profound respiratory distress due to complete agenesis of the left lung, pulmonary artery, and bronchus. Given the absence of a surgically correctable cardiac lesion and the high perioperative risk associated with his pulmonary anatomy, he was managed supportively with escalation of respiratory care and multidisciplinary coordination to optimize stability and quality of life. 

## Conclusions

This case series contributes to the growing understanding of heterotaxy syndrome by offering detailed, phenotype-driven comparisons across three cases with differing isomerism subtypes and clinical courses. While prior literature has broadly described heterotaxy-associated anomalies, few case series provide side-by-side clinical, surgical, and developmental perspectives anchored in real-world outcomes. The inclusion of a case with pulmonary vascular agenesis, a rare and severe extracardiac manifestation, adds further nuance to the known phenotypic spectrum. These cases highlight the limitations of traditional classification systems and underscore the value of individualized care planning grounded in precise anatomic and physiologic assessment. Moving forward, improved integration of imaging, genetic testing, and long-term outcome data will be essential for refining prognostication and tailoring interventions. As molecular diagnostics evolve, coupling genotype with comprehensive clinical phenotyping may offer a more accurate framework for diagnosis, risk stratification, and early intervention in this complex syndrome.
